# Evaluating Feature-Based Homography Pipelines for Dual-Camera Registration in Acupoint Annotation

**DOI:** 10.3390/jimaging11110388

**Published:** 2025-11-01

**Authors:** Thathsara Nanayakkara, Hadi Sedigh Malekroodi, Jaeuk Sul, Chang-Su Na, Myunggi Yi, Byeong-il Lee

**Affiliations:** 1 Industry 4.0 Convergence Bionics Engineering, Pukyong National University, Busan 48513, Republic of Korea; thathsara@pukyong.ac.kr (T.N.); hadi_sedigh@pukyong.ac.kr (H.S.M.); 2 Gwangju Korean Medicine Hospital, Dongshin University, Gwangju 61619, Republic of Korea; sjuomd@hanmail.net; 3 Department of Diagnostics & Acupuncture, College of Korean Medicine, Dongshin University, Naju 58245, Republic of Korea; csna@dsu.ac.kr; 4 Major of Biomedical Engineering, Division of Smart Healthcare, Pukyong National University, Busan 48513, Republic of Korea; 5 Major of Human Bioconvergence, Division of Smart Healthcare, Pukyong National University, Busan 48513, Republic of Korea

**Keywords:** infrared imaging (IR), acupoint localization, dual-camera system, feature matching, image registration

## Abstract

Reliable acupoint localization is essential for developing artificial intelligence (AI) and extended reality (XR) tools in traditional Korean medicine; however, conventional annotation of 2D images often suffers from inter- and intra-annotator variability. This study presents a low-cost dual-camera imaging system that fuses infrared (IR) and RGB views on a Raspberry Pi 5 platform, incorporating an IR ink pen in conjunction with a 780 nm emitter array to standardize point visibility. Among the tested marking materials, the IR ink showed the highest contrast and visibility under IR illumination, making it the most suitable for acupoint detection. Five feature detectors (SIFT, ORB, KAZE, AKAZE, and BRISK) were evaluated with two matchers (FLANN and BF) to construct representative homography pipelines. Comparative evaluations across multiple camera-to-surface distances revealed that KAZE + FLANN achieved the lowest mean 2D error (1.17 ± 0.70 px) and the lowest mean aspect-aware error (0.08 ± 0.05%) while remaining computationally feasible on the Raspberry Pi 5. In hand-image experiments across multiple postures, the dual-camera registration maintained a mean 2D error below ~3 px and a mean aspect-aware error below ~0.25%, confirming stable and reproducible performance. The proposed framework provides a practical foundation for generating high-quality acupoint datasets, supporting future AI-based localization, XR integration, and automated acupuncture-education systems.

## 1. Introduction

Acupuncture, a fundamental component of Korean Traditional Medicine (KTM), has demonstrated long-standing therapeutic efficacy across various medical conditions [1,2]. This treatment involves inserting fine needles through the skin into specific acupoints on the body to influence the flow of energy, known as qi, and promote healing [3]. Recent studies suggest that acupuncture can help reduce symptoms of Parkinson’s disease by improving motor function, decreasing tremors, and enhancing patients’ quality of life. These benefits are thought to arise from acupuncture-induced stimulation of neural pathways and modulation of neurotransmitter levels, providing a complementary approach to managing disease progression and symptoms observed in Parkinson’s disease [4]. Accurate localization of acupoints is essential for both effective treatment and robust research methodologies.

**Figure 1:**
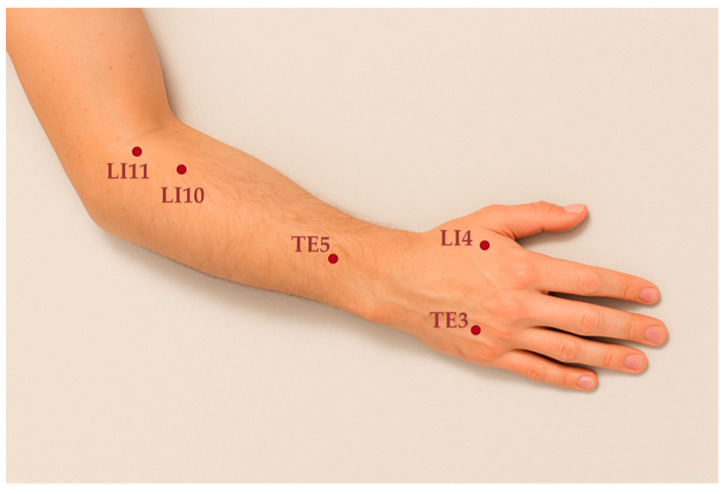
Five common acupoints on the hand (LI4, TE3, TE5, LI10, LI11).

As we move into an era of artificial intelligence (AI) and extended reality (XR), medical education and research are transforming rapidly [5]. Acupuncture, like many other medical disciplines, is being reshaped by AI. XR-based systems for acupoint education rely on precise machine learning (ML) models, which require high-quality labeled datasets. Meanwhile, XR is increasingly utilized in medical education, providing interactive learning environments that facilitate an understanding of complex anatomical structures, including acupoints. However, such AI, XR-based systems depend on precise and accurate ML models to work seamlessly in acupuncture [6,7,8,9,10]. These models must accurately classify and localize points in the human body that correspond to acupoints. Such classification and localization are highly dependent on high-quality curated data. To ensure the reliable identification of acupoints across individuals, diverse and precisely labeled datasets must be developed, providing anatomical mapping and labeling for each acupoint. Minor deviations in point localization can lead to significant inaccuracies in treatments or analysis, underscoring the importance of accuracy in these technological models. However, current data collection methods for acupuncture face challenges in terms of accuracy, consistency, and efficiency, which hinder the development of robust ML models, as well as the development of reliable models for acupuncture. In the absence of precise data collection, data-driven models are often inadequately trained, leading to outcomes that are limited and not reliably reproducible [11,12]. In this study, the authors focus on five commonly used acupoints on the hand: LI4 (Hegu), TE3 (Zhongzhu), TE5 (Waiguan), LI10 (Shousanli), and LI11 (Quchi) (see Figure 1) [2,11,13,14,15,16].

In acupuncture AI-related research, data collection often relies on conventional manual annotation, where trained professionals mark acupoints on images of the body. This approach has been used in several recent studies [11,15,17,18,19]. Despite its valuable contributions to the field, it has its limitations. Typically, software such as LabelMe and COCO annotator are used for annotation [11,15,17]. This task poses a challenge, even for experts, due to the varying appearance of the arm across different image angles. Additionally, the annotation points may shift depending on these angles.

**Figure 2:**
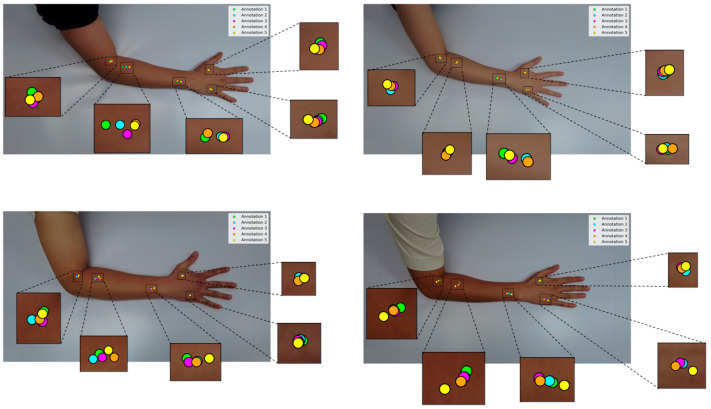
Comparison of acupoint annotations using the conventional method performed by an expert.

An experiment was conducted by a Korean acupuncture expert who annotated four different arm images five times using the conventional method, as shown in Figure 2 [11], which revealed significant variation in point localization for a single posture across multiple hand captures. When the experiments were extended to different postures, the variation became even more pronounced. Such inconsistencies can be attributed to several factors, including human error, the annotator’s subjective interpretation of 2D imagery, and limitations inherent in the sequential workflow of image acquisition followed by conventional manual annotation. Moreover, to capture a range of postures, the method necessitates repeated annotations for the same participant across separate images, thereby increasing time demands and localization variability. These issues collectively underscore the limitations of relying on conventional image annotation as a primary data source for ML model development, emphasizing its inefficiency and susceptibility to inconsistencies, particularly in specialized domains such as acupuncture annotation, where precise anatomical localization is crucial for therapeutic accuracy.

To address these limitations in data annotation and acupoint localization, we propose a novel, low-cost dual-camera imaging system integrating both IR and visible light using Raspberry Pi hardware. Unlike conventional annotation approaches, our system uses IR ink to non-invasively mark acupoints directly on the skin, which are only visible under IR light. This setup enables the simultaneous capture of both IR and visible light streams, thereby improving consistency and reducing human-induced variability.

Accurate dual-camera registration is a crucial requirement in multimodal imaging systems. Dual-camera and multimodal imaging setups have gained significant traction in medical imaging, leveraging complementary information from different spectral domains. In particular, the fusion of visible light and IR imaging has been explored to enhance diagnostic and monitoring capabilities. González-Pérez et al. [20] developed a low-cost dual-camera system integrating co-aligned thermal IR and RGB sensors for diabetic foot ulcer monitoring. In neurosurgical guidance, Müller et al. [21] combined visible-light and thermographic cameras to overlay vascular thermal maps directly onto the surgeon’s view. Ma et al. [22] provided a comprehensive review of IR and visible image fusion methods, emphasizing the rapid advancement of multimodal registration techniques over the past decade.

In the acupuncture dual-camera system, to ensure accurate registration between the IR and RGB image pairs captured by the two cameras, five state-of-the-art feature-based homography methods were evaluated, namely SIFT (Scale-Invariant Feature Transform), ORB (Oriented FAST (Features from Accelerated Segment Test) and Rotated BRIEF), KAZE, AKAZE (Accelerated-KAZE), and BRISK (Binary Robust Invariant Scalable Keypoints). These techniques, which are widely used in computer vision applications such as object recognition, image registration, and 3D reconstruction, were evaluated for their ability to accurately fuse acupoint information across modalities.

SIFT [23], introduced by Lowe, is renowned for its robustness under scale and rotation variations, making it a go-to method for high-precision applications. On the other hand, binary descriptor-based methods, such as ORB [24] and BRISK [25], strike a balance between speed and matching accuracy, which are essential for real-time applications. The integration of matches, such as FLANN (FAST Library for Approximate Nearest Neighbors) [26] and BF (Brute-Force Matcher) [27] further accelerates the process, allowing efficient handling of large feature datasets in scenarios ranging from real-time video processing to large-scale 3D mapping. Comparative studies have highlighted distinct trade-offs among these methods. While SIFT offers exceptional matching robustness, its computational expense can limit its practicality for time-sensitive tasks [23]. In contrast, ORB and BRISK deliver faster computation speeds with an acceptable trade-off in feature distinctiveness [24,25], making them popular choices for mobile applications and real-time systems, such as those used in augmented reality applications. Moreover, advancements in nonlinear scale space methods, as demonstrated by its accelerated variant AKAZE, have improved feature detection accuracy while reducing computational overhead [28,29]. When paired with efficient matching techniques like FLANN, these advanced methods facilitate rapid and reliable feature correspondence, proving their utility in a wide range of practical applications [30,31,32].

Feature-based registration techniques remain fundamental for aligning images acquired from different sensors or viewpoints. Classical feature detectors and descriptors have been widely utilized in various registration tasks. A recent comparative study evaluated six of these algorithms, SIFT, SURF, KAZE, AKAZE, ORB, and BRISK, under variations in scale, rotation, and perspective. Feature matching was refined using the Nearest-Neighbor Distance Ratio (NNDR) and RANSAC (Random Sample Consensus), demonstrating that SIFT and BRISK yielded the highest accuracy, while ORB and BRISK were the most efficient [32]. Sieler et al. [33] compared several feature detectors in intraoperative brain-surgery videos to enable motion-compensated registration, assessing spatial feature density and temporal robustness across sequential frames. Their results showed that KAZE produced the densest and most temporally stable features, outperforming other detectors in dynamic surgical environments. Similarly, Adhikari et al. [34] evaluated four feature-based methods, ORB, BRISK, KAZE, and AKAZE, combined with three outlier rejection algorithms: RANSAC, Graph-Cut RANSAC (GC-RANSAC), and Marginalizing Sample Consensus (MAGSAC++). The quantitative results demonstrated that BRISK coupled with MAGSAC++ achieved the most accurate and stable registration, exhibiting strong robustness against deformation, staining variation, and noise in histopathological slide images. In precision agriculture, Forero et al. [30] compared multiple feature-based registration algorithms (SIFT, SURF, ORB, BRISK, KAZE, and AKAZE) for aligning near-infrared (NIR) and visible images of rice crops. They found that the green channel produced the most accurate matches, while the FAST–BRISK combination offered the best performance in both speed and accuracy, confirming its effectiveness for cross-spectral agricultural imaging.

Other studies have also optimized feature-based techniques for stereo vision and aerial sensing. Yan et al. [35] proposed a stereo-vision obstacle detection system that integrates fuzzy logic and neural networks, where an improved FAST algorithm enhances illumination adaptability for real-time object detection in agricultural fields. Cowan et al. [36] investigated multiple detector–descriptor configurations for UAV (Unmanned Aerial Vehicle) guidance and navigation, highlighting the balance between robustness and real-time performance. Together, these works highlight the importance of selecting efficient, invariant, and computationally viable feature-extraction methods for multi-sensor and cross-spectral registration applications, principles that underpin the feature-matching approach used in the present dual-camera acupoint registration framework.

To achieve reliable registration between the IR and RGB image pairs, five representative feature-based homography pipelines were systematically evaluated. Floating-point descriptors such as SIFT, KAZE, and AKAZE were paired with the FLANN matcher to enable efficient approximate nearest-neighbor search in high-dimensional descriptor spaces. In contrast, binary descriptors such as ORB and BRISK were matched using the lightweight BF matcher based on the Hamming distance. The detector–matcher pairings were carefully selected to ensure algorithmic compatibility and to maintain computational feasibility on the resource-constrained Raspberry Pi 5 platform.

The study lies in the development of a low-cost dual-camera registration framework that integrates IR and RGB imaging on an embedded Raspberry Pi 5 platform to achieve accurate and reproducible acupoint localization. Unlike previous approaches that rely on conventional 2D annotation or single-camera imaging, the proposed system introduces a non-invasive IR-ink-based marking method to ensure consistent acupoint visibility across individuals, a feature-based homography pipeline for precise alignment, and a portable on-device registration implementation that operates entirely on embedded hardware. These contributions collectively provide a practical and reproducible foundation for generating high-fidelity acupoint datasets, thereby facilitating AI-based localization.

## 2. Materials and Methods

This section outlines the comprehensive methodology employed in the study, encompassing system setup, image acquisition, feature extraction, robust registration, and error evaluation. The objective was to reliably register images from dual cameras (RGB and IR) for precise mapping of acupoints.

### 2.1. Dual-Camera System Design

An infrared (IR) ink pen (LDP LLC, Carlstadt, NJ, USA) was used to mark the acupoints. The pen contains an alcohol-based (ethanol) IR ink that appears invisible on white paper but exhibits a faint green hue on the tip of the pen. The ink absorbs light at 793 nm and emits at 840 nm, making it detectable under IR light. Additionally, a fluorescent dye (D-122, BioActs, Incheon, Republic of Korea) was incorporated to enhance visibility under specific lighting conditions. D-122 was dissolved in ethanol at a concentration of 1 mg/mL and has an excitation wavelength of 774 nm with an emission wavelength of 806 nm.

The camera system was designed as a stereo setup for precise identification of acupoints, capturing both RGB and IR images. The RGB camera utilized the Raspberry Pi Camera Module v2 (8-megapixel, Raspberry Pi Foundation, Cambridge, UK) [37], known for its high-resolution image capture. For IR imaging, the NOIR Raspberry Pi Camera Module v2 (8-megapixel, Raspberry Pi Foundation, UK) [37] was incorporated. Each camera was configured to operate at a resolution of 1640 × 1232 pixels (px).

**Figure 3:**
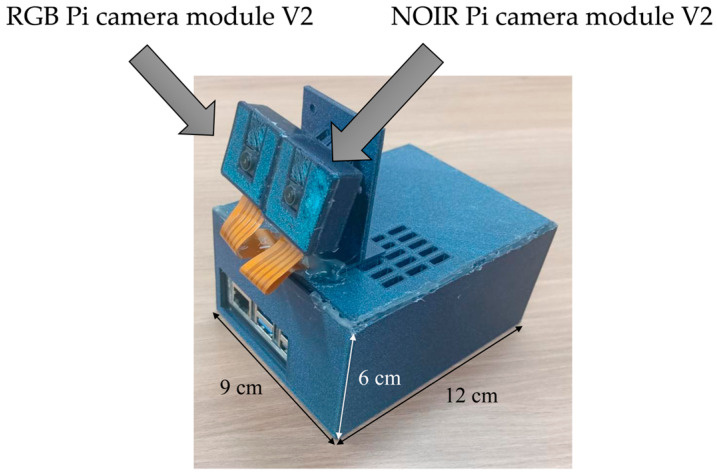
Dual-camera system design: Compact enclosure featuring RGB and NOIR cameras.

To ensure compatibility between the emission frequencies of the IR ink and the fluorescent dye, the selected NOIR camera was chosen to match their emission ranges, thereby optimizing its ability to capture accurate IR data. To balance performance and cost, the Raspberry Pi 5 module (8 GB RAM, Raspberry Pi Foundation, Cambridge, UK) [38] was selected as the development platform. This model is powered by a Broadcom BCM2712 2.4 GHz quad-core 64-bit Arm Cortex-A76 CPU, featuring cryptographic extensions, a 512 KB per-core L2 cache, and a 2 MB shared L3 cache. Although higher-resolution cameras could provide more detailed imaging, factors such as processing power requirements for real-time high-resolution video, equipment costs, and overall system complexity were carefully considered when selecting the hardware. A compact design for the camera enclosure was developed to resolve an initial setup issue that caused the camera cable to become dislodged during movement (see Figure 3). The design was created using SolidWorks software (2022 version) and fabricated via 3D printing, ensuring stability and ease of use during experiments.

Lighting conditions and viewing angles can significantly impact the accuracy of point detection in the proposed imaging system. To ensure optimal compatibility between the absorption frequencies of the IR ink and fluorescent dye, two types of IR emitters were employed, both operating at a peak wavelength of 780 nm: SMD-type IR LEDs (EPIGAP-OSA, Berlin, Germany) and axial-type IR LEDs (MTE3278N-WRC, Latham, NY, USA). Given the critical role of illumination in detection accuracy, a series of experiments was conducted to optimize the performance of the lighting system. These experiments aimed to achieve uniform and stable illumination to enhance overall image quality and improve the visibility and detection precision of acupoints.

**Figure 4:**
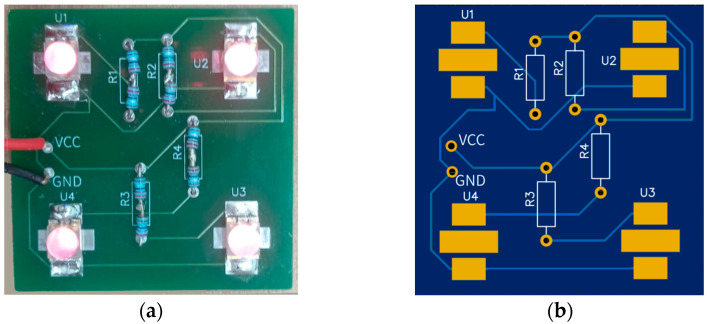
PCB designs for two IR LED emitter systems: (**a**) PCB of SMD-Type IR LED emitter system; (**b**) Schematic diagram of IR LED emitter system.

The light array was constructed by grouping four LEDs into one set and assembling four such sets to complete the emitter system (see Figure 4). To enhance light distribution, a total of eight LEDs were used. Each set incorporated reflectors to direct and disperse the light efficiently, while diffuser sheets were added to ensure smooth and uniform illumination.

### 2.2. Selection of an Appropriate Acupoint Marking Method

A series of experiments was conducted to develop an accurate and reliable acupoint identification and data collection system by identifying the most practical combination of ink or fluorescent dye, camera system, and light source. The primary objective was to optimize the visibility and precision of acupoint markings. The experiments systematically varied the imaging configuration using a custom-designed dual-camera system alongside an industrial IR-sensitive camera (Alvium 1800 U-895, Stadtroda, Germany). An IR ink pen and fluorescent dyes with multiple ethanol dilutions were evaluated under different emitter wavelengths (780 nm and 850 nm) on various test surfaces, including white paper, laminated paper, and human skin.

### 2.3. Stereo Vision-Based Point Registration and Feature Matching

**Figure 5:**
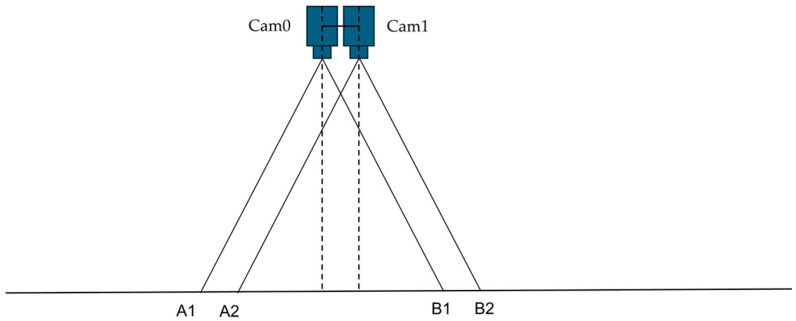
Dual-camera system setup and overlapping field of view.

**Figure 6:**
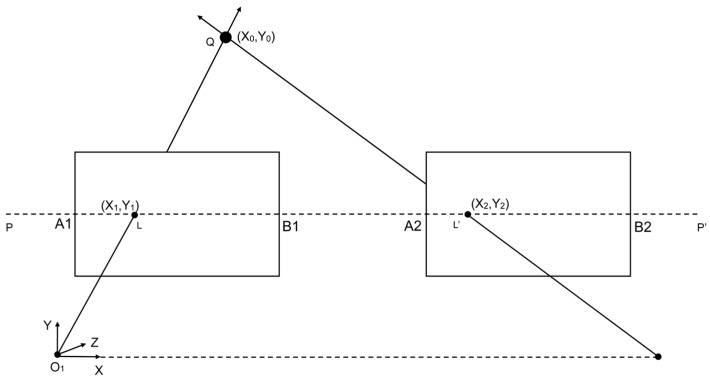
Projection geometry of the stereo camera system.

To establish precise projection geometry for the dual-camera system, the cameras were carefully positioned and calibrated to achieve optimal registration. Figure 5 and Figure 6 illustrate the arrangement and working principles of the stereo camera setup, highlighting the projection geometry and registration process.

**Figure 7:**
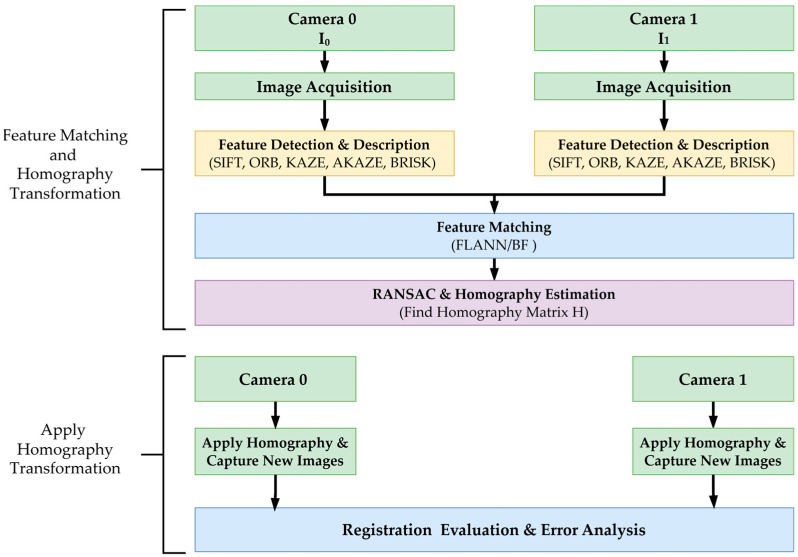
The dual-camera image registration during the feature matching and homography estimation process.

Figure 5 and Figure 6 illustrate our dual-camera arrangement, where the cameras are mounted in parallel along a shared horizontal axis, separated by a small baseline of 3 cm. This arrangement, consisting of Cam 0 and Cam 1, captures overlapping ground areas A1B1 and A2B2. Each camera’s optical axis in this configuration is oriented downward, focusing on the same plane for data acquisition. A generic point Q in the scene, specified by (x_0_, y_0_) in the global coordinate system, projects onto the image planes of Cam 0 and Cam 1 at (x1, y1) and (x2, y2), respectively. The line $p-p^{\prime }$ denotes a reference for the two camera planes. Meanwhile, O_1_ marks the origin of the 3D coordinate system, with the X axis extending horizontally, the Y axis vertically, and the Z axis perpendicular to the plane. By leveraging these known geometric relationships, corresponding features from both cameras can be identified and aligned, forming the foundation of the feature-matching methodology described in the following sections. Figure 7 illustrates the dual-camera image registration process, which utilizes feature matching and homography estimation.

This setup enables direct comparison of feature-matching performance between the two image views by ensuring precise registration. The captured images serve as input for subsequent feature detection and matching. Homography is necessary for this process to align the images from both cameras into a standard coordinate frame, correcting for perspective distortions and ensuring accurate feature correspondence [39].

Feature detection aims to identify distinct and repeatable keypoints within an image, while feature description characterizes these keypoints for robust matching across different views. This study evaluated five feature detection and description methods (SIFT, ORB, KAZE, AKAZE, and BRISK) in terms of their performance.

Each method follows a standard two-step process: keypoints are detected based on key intensity variations, and other image characteristics and descriptors are computed to represent the local image appearance. In the SIFT method, keypoints are detected by identifying extrema in a Difference of Gaussian (DoG) pyramid [23], and a 128-dimensional descriptor vector represents each keypoint:

(1) $$d\;=\;\left[d_{1},d_{2},........,d_{128}\right]$$

where each $d_{i}$ represents the gradient orientation histogram over a local image patch. Other methods, such as ORB, utilize the FAST detector for keypoint identification and generate binary descriptors using the BRIEF algorithm [40]. Similarly, KAZE and AKAZE construct a nonlinear scale-space to detect keypoints, with AKAZE providing an additional option for binary descriptors [28,29]. BRISK relies on intensity variations within a circular sampling pattern to detect keypoints and generate binary descriptors [25]. After detecting and describing keypoints, features are matched across the two camera images.

Two matching strategies were used depending on the descriptor type (FLANN vs. BF). Lowe’s ratio test is applied to improve the quality of matches [23]. In this test, the distance of the closest match m is compared with that of the second-closest match n using a ratio where τ is a threshold typically set to 0.7:

(2) $$\frac{n}{m}\;<\;\;\tau$$

This threshold effectively filters out ambiguous matches by ensuring that the best match is significantly closer than the second-best option [23]. Five matching approaches were evaluated: SIFT + FLANN, ORB + BF, AKAZE + FLANN, KAZE + FLANN, and BRISK + BF. Detector matcher pairings were selected based on descriptor type compatibility, on-device computational constraints, and the need for a fair cross-family performance comparison. Floating-point descriptors (SIFT, KAZE, AKAZE) were paired with FLANN, which is optimized for high-dimensional continuous feature spaces and provides efficient approximate nearest-neighbor searches with consistent recall. In contrast, binary descriptors (ORB, BRISK) were paired with the BF matcher using the Hamming distance metric, which directly leverages bitwise comparisons without requiring conversion to floating-point vectors. This configuration ensures predictable latency on computationally limited embedded platforms such as the Raspberry Pi 5 while maintaining methodological fairness across descriptor families.

The matched keypoints are used to compute a 3 × 3 homography matrix **H**, which aligns points from Camera 0 to Camera 1, where $\left(x_{0},y_{0}\right)$ is a point in the image from Camera 0 and ${(x}_{1},y_{1})$, the corresponding point in Camera 1 after transformation. Homography estimation is performed using RANSAC to filter out incorrect match errors [41]. The homography matrix **H** is obtained by minimizing the transformation error, where only the inlier matches contribute to the estimation. The inlier ratio, defined as the number of inlier matches divided by the total number of suitable matches, indicates the robustness of the feature matching process.

(3) $$\left[\begin{array}{c} x_{1}\\ y_{1}\\ 1 \end{array}\right]=H\;\left[\begin{array}{c} x_{0}\\ y_{0}\\ 1 \end{array}\right]=\left[\begin{array}{ccc} h_{11} & h_{12} & h_{13}\\ h_{21} & h_{22} & h_{23}\\ h_{31} & h_{32} & h_{33} \end{array}\right]\;\left[\begin{array}{c} x_{0}\\ y_{0}\\ 1 \end{array}\right]$$

(4) $$H={argmin}_{H}\;{\sum_{i=1}^{N}\left\|x_{0,i}-Hx_{i}\right\|}^{2}$$

The inlier ratio was not predefined but was automatically computed after RANSAC-based homography estimation using a reprojection threshold of 5 px. This value represents the proportion of geometrically consistent correspondences used to estimate H. To ensure a sufficient number of reliable matches for stable homography estimation, high-resolution images (1640 × 1232 px) were used together with dense feature detectors and Lowe’s ratio test to retain only strong and spatially well-distributed correspondences. While the homography matrix is computed with suitable matches, the inlier count is measured to evaluate the inlier ratio and average computational cost per image pair. The inlier ratio measures the percentage of matched keypoints consistent with the estimated homography, indicating the method’s robustness against outliers. The average computational cost per image pair is recorded to assess the feasibility of each technique for real-time applications. Once the homography matrix is computed, it is applied to transform and align new images captured from Camera 0 with those from Camera 1. The effectiveness of each feature matching-based camera calibration is evaluated based on registration accuracy metrics.

Registration accuracy is evaluated by calculating the error between manually annotated ground truth points and their corresponding transformed points. Let $\left(x_{i},\;{\;y}_{i}\right)$ represent the ground truth coordinates and $\left(x_{i}^{t},\;{\;y}_{i}^{t}\right)$ denote the transformed coordinates for N corresponding points. The horizontal and vertical errors are defined as the differences between the respective coordinates along the x and y axes (Equation (5)). The percentage errors along the x and y axes are calculated as the relative deviations between the ground truth and transformed points (Equation (6)). The mean 2D error in pixels is then calculated as the average of these errors across all N points (Equation (7)). Finally, the mean aspect-aware error, which normalizes the errors concerning the image dimensions, is defined (Equation (8)).

(5) $${∆x}_{i}(px)=\left|x_{i}-x_{i}^{t}\right|,\;{∆y}_{i}(px)=\left|y_{i}-y_{i}^{t}\right|$$

(6) $$Error,x(\%)=\frac{∆x}{Width}\times 100\%\;,\;\;\;\;\;\;\;\;\;\;\;\;Error,y(\%)=\frac{∆y}{Height}\times 100\%$$

(7) $$Mean\;2D\;Error\;(px)=\frac{1}{N}\sum_{i=0}^{N}\sqrt{{\left(∆x_{i}\right)}^{2}+{\left(∆y_{i}\right)}^{2}}$$

(8) $$Mean\;Aspect\;Aware\;Error\;(\%)=\frac{100}{N}\sum_{i=0}^{N}\sqrt{{\left(\frac{∆x_{i}}{\;Width}\right)}^{2}+{\left(\frac{∆y_{i}}{Height}\right)}^{2}}$$

### 2.4. Stereo Vision-Based Acupoint Localization

**Figure 8:**
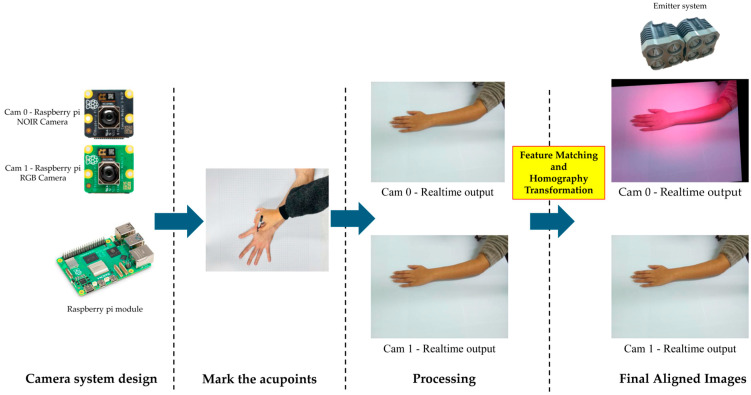
Proposed pipeline for acupuncture data collection.

**Figure 9:**
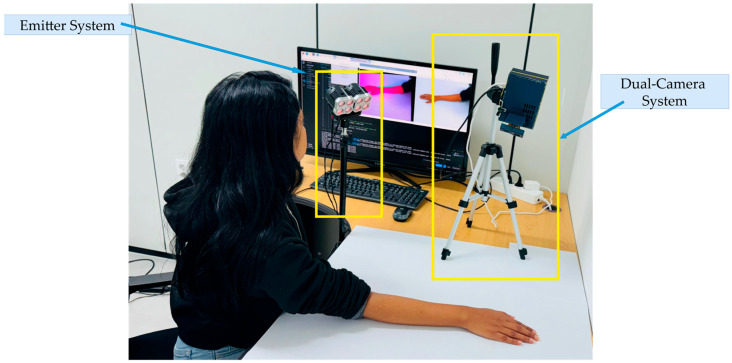
Dual-camera and emitter system setup for acupoint annotation.

Our proposed pipeline for detecting acupoints utilizes a dual-camera system and an IR emitter setup. As shown in Figure 8, the pipeline begins by marking acupoints using an IR ink pen. The process is simultaneously captured by the NOIR camera (Cam 0) and the RGB camera (Cam 1). A homography transformation is applied onboard a Raspberry Pi to produce final, aligned images. As illustrated in Figure 9, the dual-camera system consists of two main components: the emitter system and the camera system. The emitter system creates an IR environment, enhancing the visibility of acupoints marked with IR ink. The camera system and emitter setup are positioned on the table, enabling the simultaneous transmission and capture of visible and IR images.

**Figure 10:**
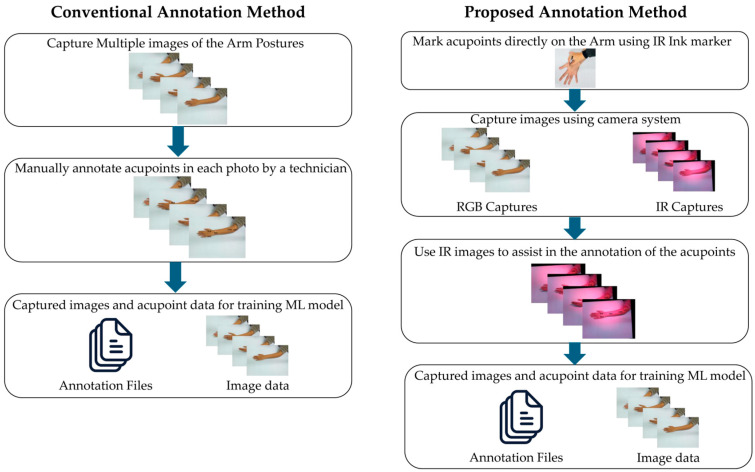
Comparison of conventional and proposed acupuncture annotation methods.

As shown in Figure 10, the conventional approach requires a technician to manually label acupoints on multiple RGB images, which are time-consuming and prone to errors. In contrast, our approach utilizes IR-based markers to streamline the annotation process, providing a more efficient and dependable method for preparing data for ML model training.

**Figure 11:**
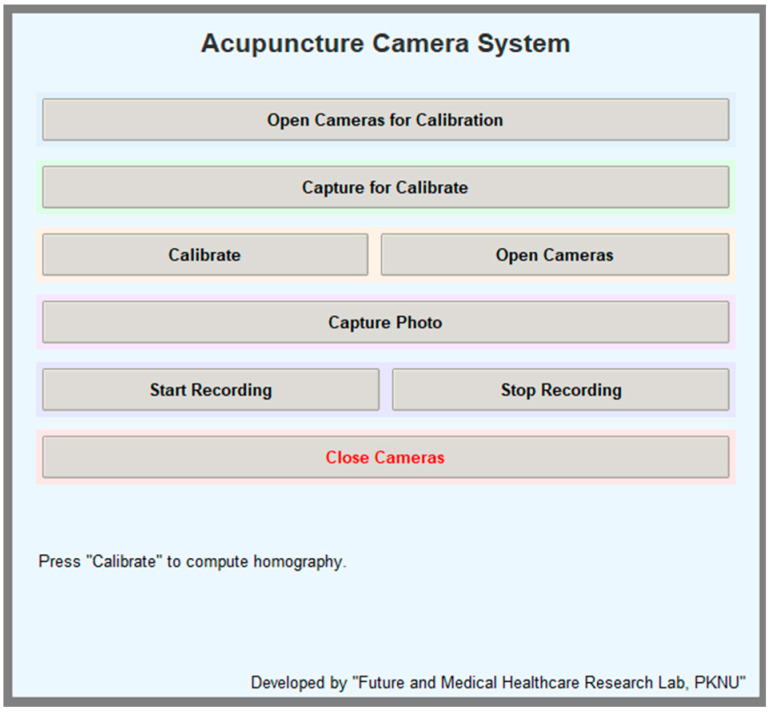
Python-based GUI for the dual-camera registration system.

To streamline the dual-camera data collection workflow, a custom graphical user interface (GUI), as illustrated in Figure 11, was developed using Python 3.9.18. The interface, implemented using the Tkinter framework, provides user-friendly control over the entire acquisition pipeline, including camera initialization, calibration capture, homography computation, and image or video recording.

### 2.5. Multi-Distance Evaluation of Feature-Based Homography Methods

To comprehensively assess registration robustness, five feature-based homography pipelines (SIFT + FLANN, ORB + BF, AKAZE + FLANN, KAZE + FLANN, and BRISK + BF) were compared using our dual-camera setup. Twenty markers were positioned at predefined locations to ensure uniform spatial coverage. The px coordinates of these twenty marker positions were extracted from both the RGB and NOIR images to evaluate spatial correspondence. The registration accuracy was quantified by computing the mean 2D displacement error between the two image planes, providing a direct measure of registration precision. To further analyze the influence of camera-to-surface distance on registration stability, identical experiments were repeated at separations of 40, 50, and 60 cm between the camera module and the planar surface. For each distance, twenty corresponding point pairs were recorded, and the mean pixel-level errors were calculated independently. This procedure enabled evaluation of each feature-matching method’s sensitivity to geometric scale variation, revealing how registration accuracy degrades or stabilizes with increased working distance.

### 2.6. Evaluation of Dual-Camera Registration Using Hand Images

To evaluate the registration accuracy of the dual-camera system under practical conditions, experiments were conducted using hand images captured simultaneously from both cameras. During image acquisition, five acupoints commonly used in traditional Korean medicine (LI11, LI10, TE5, TE3, and LI4) were selected, as described in Figure 1. Data were collected from twelve participants across four distinct hand postures to comprehensively assess the accuracy of acupoint registration. Small sticker markers were applied to indicate the location of each acupoint, and images were captured using both RGB and IR cameras. Subsequently, homography-based transformation was applied to align the paired camera images, enabling pixel-wise correspondence analysis between modalities. Finally, all acupoints were manually annotated using the COCO Annotator (v 0.11.1) to obtain precise ground-truth coordinates for quantitative evaluation of registration error.

### 2.7. Evaluation of Annotation Consistency in RGB and IR Imaging

To evaluate the consistency and reliability of acupuncture-point annotation across different imaging modalities, simultaneous captures were obtained from both the RGB and NOIR cameras under four distinct arm postures. The captured image sets were subsequently annotated independently by three technicians using the COCO Annotator. This experiment aimed to assess inter-annotator variability between the conventional RGB-based labeling process and the proposed IR ink-assisted method. In the RGB condition, acupoints were manually located based on visible anatomical landmarks. In contrast, in the IR condition, the same points were pre-marked with IR ink that became clearly distinguishable under IR light. By comparing these two annotation modalities, the study examined how the enhanced visibility of IR-marked acupoints influences annotation precision, reduces subjective interpretation errors, and improves reproducibility in dataset preparation for AI-based acupoint localization.

## 3. Results

### 3.1. Marking Materials Performance Comparison

In this study, as described in Section 2.2, the performance of the IR ink and fluorescent dyes (with varying ethanol dilutions) was evaluated across three experimental conditions using different camera systems and emitter wavelengths. Our results demonstrated that the best marker clarity and contrast performance was achieved using the designed camera system with a 780 nm emitter and an IR ink that exhibits strong absorption at 793 nm and emission at 840 nm. These findings informed the subsequent registration process, ensuring that our system could effectively translate marker visibility into precise spatial correspondence. Supplementary Materials Table S1 summarizes the quantitative results, highlighting that a close spectral match between the emitter wavelength and the absorption peaks of the marking materials leads to higher brightness and contrast on white paper, laminated paper, and human skin. Conversely, increasing the ethanol dilution consistently reduced the fluorescence intensity, although all samples remained detectable. Supplementary Figures S1–S5 provide representative images illustrating these results.

### 3.2. Multi-Distance Feature-Based Homography Methods Performance

Table 1 summarizes the registration performance of the five feature-based homography pipelines across three camera-to-surface distances (40 cm, 50 cm, and 60 cm). At 40 cm, all methods achieved stable registration accuracy, with mean 2D errors ranging from ~1.78 px (KAZE + FLANN) to ~2.37 px (SIFT + FLANN). KAZE + FLANN consistently exhibited the lowest mean 2D error (1.78 ± 1.13 px) and the smallest mean aspect-aware error (0.13 ± 0.08%), demonstrating strong robustness to perspective distortion at short working distances. When the camera-to-surface distance increased to 50 cm, the overall accuracy improved slightly, reflecting a reduction in parallax distortion in the mid-range configuration. KAZE + FLANN maintained the top performance with a mean 2D error of 1.29 ± 0.83 px and mean aspect-aware error of 0.09 ± 0.06%, followed closely by AKAZE + FLANN (1.36 ± 0.64 px). At 60 cm, registration accuracy remained stable across methods. KAZE + FLANN again demonstrated the best performance, achieving a mean 2D error of 1.17 ± 0.70 px and the mean aspect-aware error of 0.08 ± 0.05%. These findings highlight that KAZE + FLANN provides reliable and precise registration performance.

**Figure 12:**
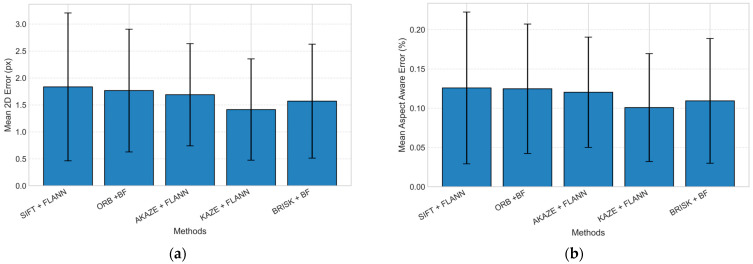
Comparison of feature-based homography pipelines averaged across all evaluated camera-to-surface distances. (**a**) Mean 2D error distribution in pixels by approach; (**b**) Mean aspect-aware error percentage by approach.

Figure 12a,b present the aggregated mean ± standard deviation values of the 2D error (px) and mean aspect-aware error (%), respectively, averaged across all evaluated camera-to-surface distances. The results show a consistent performance trend across methods, confirming that registration accuracy remained stable within the tested range. Among all pipelines, KAZE + FLANN achieved the lowest overall mean 2D error and the lowest aspect-aware error. BRISK + BF and AKAZE + FLANN exhibited slightly higher errors, whereas SIFT + FLANN and ORB + BF showed larger deviations.

### 3.3. Performance of Dual-Camera Registration Using Hand Images

**Figure 13:**
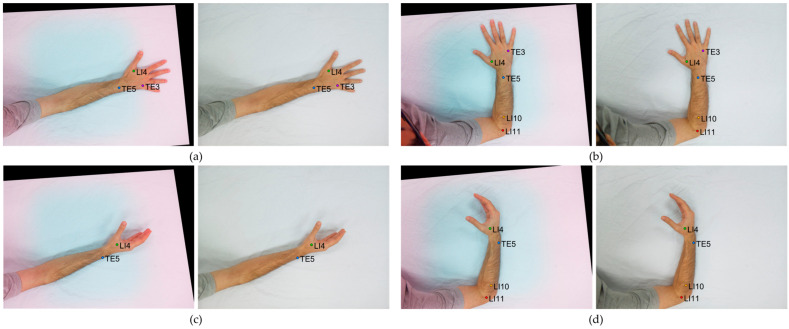
Hand postures are used for dual-camera registration evaluation. Each pair shows simultaneous NOIR (**left**) and RGB (**right**) camera captures. (**a**) Posture A—Arm extended, palm down; (**b**) Posture B—Arm bent, palm side; (**c**) Posture C—Arm extended, palm inward; (**d**) Posture D—Arm bent, palm side.

The dual-camera system was calibrated using the KAZE + FLANN feature-matching method, which demonstrated the highest comparative registration accuracy in the multi-distance evaluations. To evaluate its practical performance under real-world conditions, data from twelve participants performing four different hand postures were used, as illustrated in Figure 13.

Data were collected from twelve participants, each performing four hand postures, yielding 48 (12 × 4) image pairs captured by both cameras. For each posture (A–D), 2D pixel errors and aspect-aware errors were computed for all visible acupoints and averaged across the 12 participants for that posture to obtain the mean and standard deviations. Table 2 presents the posture-wise (A–D) mean 2D error and aspect-aware error for the five acupoints, with each value averaged across the 12 image pairs for each posture. Overall, the dual-camera system demonstrated stable registration, with the mean aspect-aware errors remaining below ~0.25% in all visible cases. The lowest mean 2D error of 1.01 ± 0.70 px was achieved for LI4 in posture B. Points such as TE3 and TE5 also demonstrated strong stability, yielding errors of 1.21 ± 0.68 px (posture A) and 1.48 ± 0.76 px (posture C), respectively. In contrast, LI4 exhibited larger deviations in posture D (2.89 ± 1.25 px). For the distal points, LI10 and LI11 obtained their lowest mean 2D errors of 1.81 ± 0.79 px and 1.76 ± 0.94 px, respectively, in posture D. These results confirm that the system calibrated with KAZE + FLANN consistently provided an error of less than ~3 px across various hand orientations. Several acupoints were marked as PNV (Point Not Visible) because not all points were visible in specific postures, particularly in postures A, C, and D.

### 3.4. Performance Evaluation of Annotation Consistency in RGB and IR Imaging

**Figure 14:**
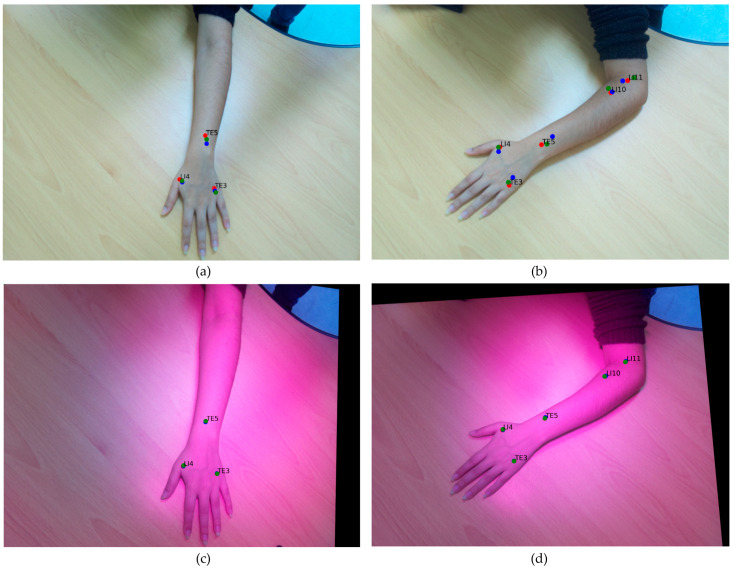
Comparison of postures A and B for acupoint annotation in RGB and IR images using conventional and IR ink marking methods. (**a**,**b**) Traditional annotation approach; (**c**,**d**) IR ink dual-camera annotation approach. Red, green, and blue dots represent the annotations made by Annotator 1, Annotator 2, and Annotator 3, respectively.

**Figure 15:**
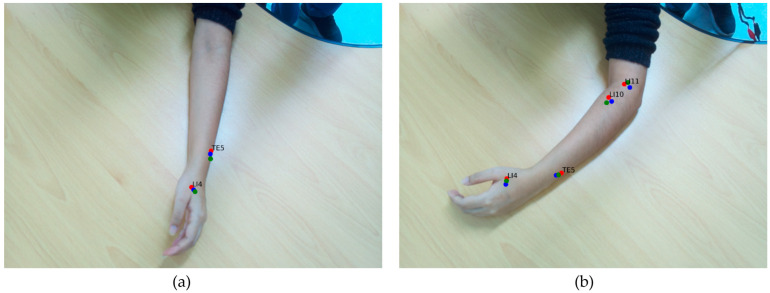

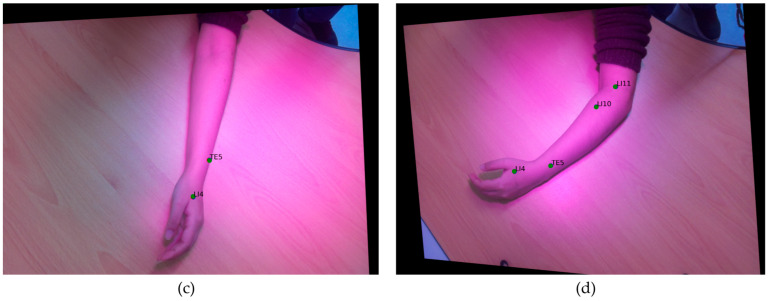
Comparison of postures C and D for acupoint annotation in RGB and IR images using conventional and IR ink marking methods. (**a,b**) Traditional approach of annotation (**c,d**), IR ink dual-camera annotation approach. Red, green, and blue dots represent the annotations made by Annotator 1, Annotator 2, and Annotator 3, respectively.

Figure 14 and Figure 15 illustrate the comparative annotation outcomes obtained from the RGB and IR image sets under four different arm postures. Three technicians independently annotated each image set. In the RGB imaging condition, the annotations exhibited considerable variation across technicians. This inconsistency arises from inherent challenges, including human perceptual errors and differing interpretations of point locations in two-dimensional RGB images. These factors undermine the precision required for training practical AI models in acupuncture applications.

Conversely, the use of IR ink resulted in markedly higher consistency and precision in the IR imaging condition. The IR ink markings, clearly visible under infrared illumination, substantially reduced ambiguity and annotation discrepancies compared with the traditional RGB approach. By leveraging the dual-camera setup to capture these IR-enhanced images, the system achieved annotation precision that significantly outperformed the conventional RGB method.

This comparison highlights that the IR ink and dual-camera approach can improve annotation repeatability compared with conventional acupoint annotation. By addressing the limitations of manual RGB annotation, this approach is crucial for advancing AI-driven tools in clinical and educational contexts.

## 4. Discussion

The proposed dual-camera system integrates IR and RGB imaging with standardized IR ink markings to enhance annotation consistency and image registration, thereby enabling accurate and reproducible localization of acupoints compared to RGB-only methods. By reducing inter and intra-annotator variability, it supports the creation of high-quality datasets for AI-based acupuncture training.

The pairing of feature detectors and matchers in this study was designed to strike a balance between algorithmic compatibility and computational feasibility on the Raspberry Pi 5 platform. Although deep learning-based keypoint detectors require accelerated computation to achieve real-time performance. Given the goal of developing a low-cost, portable, and reproducible embedded imaging system, this study deliberately focused on handcrafted feature descriptors that can operate entirely on-device without pre-training. Future research could integrate lightweight deep feature extractors once acupoint-specific annotated datasets become sufficiently large and comprehensive.

The five feature-based homography pipelines exhibited processing times ranging from approximately 0.25 s to 3.2 s per frame. The system achieved a feature-matching rate of up to 4 frames per second (fps), which is adequate for sequential image registration and manual annotation workflows but remains insufficient for fully automated or continuous processing. This level of responsiveness meets the requirements of offline data collection tasks, where images are captured frame by frame rather than through constant video streams. However, for real-time or depth-assisted applications such as continuous acupoint tracking or automatic calibration during hand movement, the current throughput becomes a limiting factor. Future improvements could include parallelized feature extraction, hardware-assisted computation or adaptive resolution sampling to enhance frame rate without sacrificing registration accuracy. Overall, the current implementation achieves a practical balance between computational efficiency and registration precision for controlled data collection, while further optimization will be essential to enable real-time automation.

The IR camera consistently detected IR ink markings across the skin types examined, demonstrating generally good performance. Detection was most pronounced on lighter or fair skin tones, where diffuse reflectance enhanced marker contrast and reduced background noise, whereas relative contrast decreased on darker skin tones. In the present setup, eight 780 nm IR LED emitters were used to provide uniform illumination of the capture area. Increasing the number or optimizing the spatial distribution of emitters could further enhance the visibility of the marker. Additional refinements, such as adaptive emitter intensity control, polarizing filters, or exposure compensation, may improve robustness across a broader range of complexions. Moreover, moderate variations in ambient lighting and minor hand motion did not cause substantial degradation in registration accuracy.

Another limitation of the current system is its dependence on manual IR ink markings, which restricts full automation. Future developments of this study could address this limitation through landmark or template-based localization, in which anatomical cues such as the wrist crease or knuckle centers are automatically detected, and a standardized acupoint atlas is adaptively aligned to the individual hand. Such approaches could substantially reduce operator involvement, enhance reproducibility, and advance the system toward marker-free, automated acupoint localization suitable for both clinical and educational applications.

## 5. Conclusions

This study introduces a cost-effective dual-camera imaging system to improve the accuracy and efficiency of acupuncture-point data collection in Traditional Korean Medicine. The developed system leverages a Raspberry Pi 5 platform, an IR ink formulation, and optimized emitter configurations to achieve precise and reproducible acupoint localization. The experimental comparisons confirmed that the IR ink provided the highest contrast and stability under IR illumination, making it the most reliable marking material for consistent acupoint detection. Among the five evaluated pipelines, KAZE + FLANN consistently demonstrated superior robustness, achieving mean 2D errors as low as 1.17 ± 0.70 px and aspect-aware errors of 0.08 ± 0.05% across multiple distances, while maintaining feasible computation on the Raspberry Pi 5 platform. In the hand-image experiments, dual-camera registration achieved mean 2D errors less than ~3 px and mean aspect-aware errors less than ~0.25%, confirming stable and reproducible performance on real, collected data. Although the minimum observed frame rates limit continuous or real-time operation, the achieved precision meets the requirements for controlled data collection in ML model training. The proposed system provides a scalable foundation for developing high-fidelity acupuncture datasets, supporting the advancement of AI-driven, XR-enabled diagnostic and educational applications in Korean traditional medicine.

## Figures and Tables

**Table 1:** Quantitative comparison of feature-based homography pipelines at three camera-to-surface distances.

Camera to Surface Distance	Method	Mean Error, X (%)	Mean Error, Y (%)	Mean 2D Error (px)	Mean Aspect Aware Error (%)
40 cm	SIFT + FLANN	0.11 ± 0.08	0.11 ± 0.11	2.37 ± 1.82	0.17 ± 0.13
	ORB + BF	0.08 ± 0.05	0.10 ± 0.09	1.90 ± 1.17	0.14 ± 0.09
	AKAZE + FLANN	0.09 ± 0.06	0.09 ± 0.09	2.06 ± 1.22	0.14 ± 0.09
	KAZE + FLANN	0.07 ± 0.06	0.10 ± 0.08	1.78 ± 1.13	0.13 ± 0.08
	BRISK + BF	0.08 ± 0.06	0.10 ± 0.10	1.90 ± 0.55	0.14 ± 0.12
50 cm	SIFT + FLANN	0.07 ± 0.06	0.06 ± 0.06	1.51 ± 1.05	0.10 ± 0.07
	ORB + BF	0.09 ± 0.08	0.10 ± 0.08	2.13 ± 1.35	0.15 ± 0.09
	AKAZE + FLANN	0.05 ± 0.04	0.06 ± 0.06	1.36 ± 0.64	0.10 ± 0.05
	KAZE + FLANN	0.04 ± 0.04	0.07 ± 0.06	1.29 ± 0.83	0.09 ± 0.06
	BRISK + BF	0.07 ± 0.04	0.06 ± 0.06	1.56 ± 0.64	0.11 ± 0.05
60 cm	SIFT + FLANN	0.07 ± 0.06	0.06 ± 0.05	1.62 ± 0.89	0.11 ± 0.06
	ORB + BF	0.05 ± 0.03	0.05 ± 0.05	1.27 ± 0.54	0.09 ± 0.04
	AKAZE + FLANN	0.05 ± 0.04	0.09 ± 0.06	1.65 ± 0.73	0.12 ± 0.06
	KAZE + FLANN	0.05 ± 0.04	0.05 ± 0.05	1.17 ± 0.70	0.08 ± 0.05
	BRISK + BF	0.05 ± 0.04	0.05 ± 0.05	1.25 ± 0.59	0.09 ± 0.04

**Table 2:** Acupoint registration error for the proposed dual-camera system under four different hand postures (A–D).

Acupoints Point	Posture A		Posture B		Posture C		Posture D	
	Mean 2D Error (px)	Mean Aspect Aware Error (%)	Mean 2D Error (px)	Mean Aspect Aware Error (%)	Mean 2D Error (px)	Mean Aspect Aware Error (%)	Mean 2D Error (px)	Mean Aspect Aware Error (%)
LI4	2.07 ± 0.85	0.14 ± 0.05	1.01 ± 0.70	0.07 ± 0.05	2.53 ± 1.09	0.16 ± 0.07	2.89 ± 1.25	0.19 ± 0.10
TE3	1.21 ± 0.68	0.08 ± 0.04	1.26 ± 0.58	0.08 ± 0.04	PNV	PNV	PNV	PNV
TE5	2.31 ± 1.15	0.15 ± 0.07	2.20 ± 0.85	0.15 ± 0.06	1.48 ± 0.76	0.10 ± 0.05	2.72 ± 1.79	0.20 ± 0.15
LI10	PNV	PNV	2.27 ± 1.25	0.16 ± 0.09	PNV	PNV	1.81 ± 0.79	0.13 ± 0.06
LI11	PNV	PNV	2.27 ± 2.04	0.16 ± 0.14	PNV	PNV	1.76 ± 0.94	0.13 ± 0.08

Note: PNV indicated as point not visible due to occlusion.

## Data Availability

The data presented in this study are available on request from the corresponding authors. The data are not publicly available due to privacy concerns.

## Supplementary Materials

The following supporting information can be downloaded at: https://www.mdpi.com/article/10.3390/jimaging11110388/s1, Figure S1: Experiment 1; Figure S2: Experiment 2; Figure S3: Experiment 3; Figure S4: Image captured using a Raspberry Pi NOIR camera under a 780 nm IR emitter system, illustrating the visibility of IR ink acupoint markings on the arm; Table S1: Visibility of IR-ink and fluorescent-dye markings across cameras/illumination settings.

## Abbreviations

The following abbreviations are used in this manuscript:

AI	Artificial Intelligence
AKAZE	Accelerated-KAZE
AR	Augmented Reality
BF	Brute-Force Matcher
BRISK	Binary Robust Invariant Scalable Keypoints
FAST	Features from Accelerated Segment Test
FLANN	Fast Library for Approximate Nearest Neighbors
KTM	Korean Traditional Medicine
LDP	Light Diffusion Pen
ORB	Oriented FAST and Rotated BRIEF
PX	Pixels
RANSAC	Random Sample Consensus
RGB	Red, Green, Blue
IR	Infrared
SIFT	Scale-Invariant Feature Transform
