# Supplementary material for: Evaluating Feature-Based Homography Pipelines for Dual-Camera Registration in Acupoint Annotation
Source: J Imaging. 2025 Nov 1;11(11):388. doi: 10.3390/jimaging11110388 (PMC12653087; doi:10.3390/jimaging11110388)
Supplement: Supplementary file 1 [file jimaging-11-00388-s001.zip › jimaging-3914067-supplementary.pdf]

Supplementary Materials

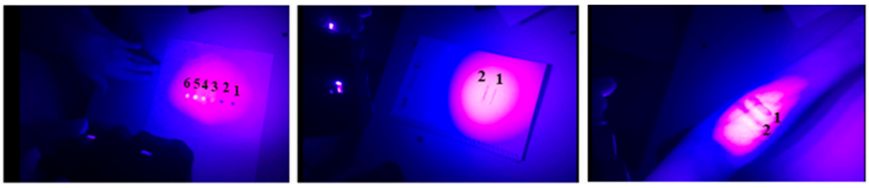

(a) (b) (c)

1 - IR ink
2 - Fluorescent dye
3 - Fluorescent dye: Ethanol = 1:1
4 - Fluorescent dye: Ethanol = 1:2
5- Fluorescent dye: Ethanol = 1:3
6 - Fluorescent dye: Ethanol = 1:4

Figure S1. Experiment 1: Raspberry NOIR camera images illustrating the fluorescence visibility of different surfaces treated with IR ink and fluorescent dye under a 780 nm emitter light system: (a) White paper, (b) Light-colored laminated paper, (c) Human skin.

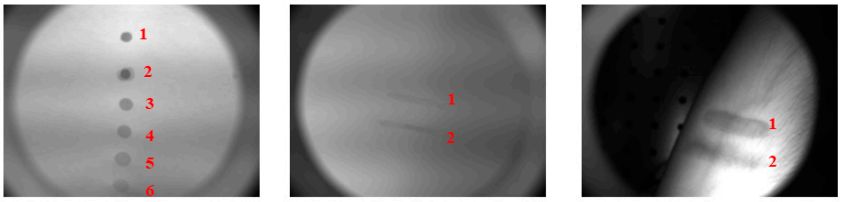

(a) (b) (c)

1 - IR ink
2 - Fluorescent dye
3 - Fluorescent dye: Ethanol = 1:1
4 - Fluorescent dye: Ethanol = 1:2
5- Fluorescent dye: Ethanol = 1:3
6 - Fluorescent dye: Ethanol = 1:4

Figure S2. Experiment 2: Industrial IR-sensitive camera (Alvium 1800 U-895) images of test surfaces treated with IR ink and fluorescent dye under a 780 nm emitter light system: (a) White paper, (b) Light-colored laminated paper, (c) Human skin.

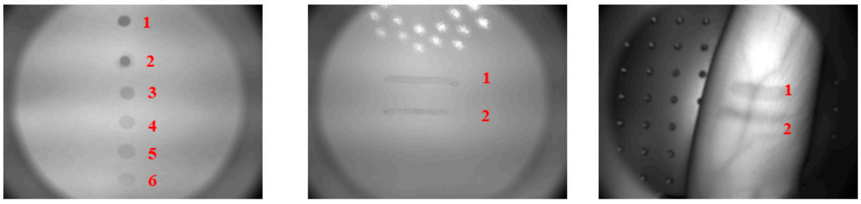

(a) (b) (c)

1 - IR ink
2 - Fluorescent dye
3 - Fluorescent dye: Ethanol = 1:1
4 - Fluorescent dye: Ethanol = 1:2
5- Fluorescent dye: Ethanol = 1:3
6 - Fluorescent dye: Ethanol = 1:4

Figure S3. Experiment 3: Industrial IR-sensitive camera (Alvium 1800 U-895) images of test surfaces treated with IR ink and fluorescent dye under the 850 nm emitter light system: (a) White paper, (b) Light-colored laminated paper, (c) Human skin.

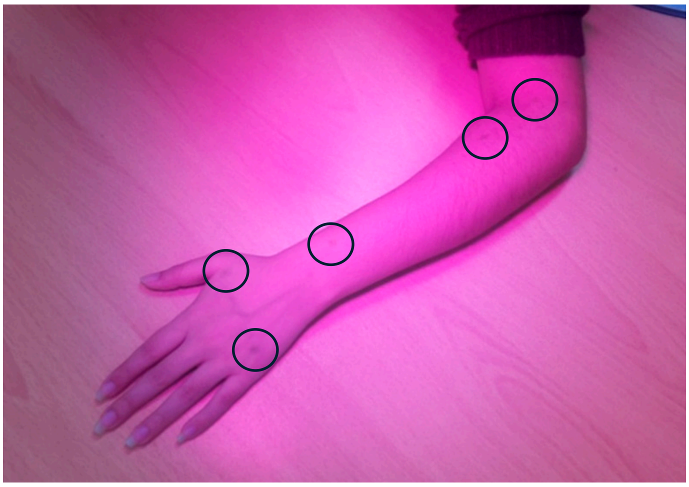

Figure S4. Image captured using a Raspberry Pi NOIR camera under a 780 nm IR emitter system, illustrating the visibility of IR ink acupoint markings on the arm. Black circular overlays indicate the detected IR ink marking positions.

Table S1. Visibility of IR-ink and fluorescent-dye markings across cameras/illumination settings.

Qualitative scale: **VH**=Very High, **H**=High, **M**=Moderate, **L**=Low, **VL**=Very Low/Faint.

No.	Marking Material	Experiment 1 (Raspberry NOIR Camera, 780 nm Illumination)	Experiment 2 (Industrial IR Camera, 780 nm Illumination)	Experiment 3 (Industrial IR Camera, 850 nm Illumination)
1	IR ink (Abs. ~793 nm; Em. ~840 nm)	Paper/Laminate & Skin: VH with sharp contrast	Paper/Laminate: VH; Skin: H (slightly lower than paper)	Paper/Laminate & Skin: H–M (off-peak; slight brightness drop)
2	Fluorescent dye (undiluted) (Abs. ~774 nm; Em. ~806 nm)	Paper/Laminate: VH; Skin: H	Paper/Laminate: H–VH; Skin: M–H	Overall: M–L; still detectable on lighter surfaces; reduced intensity on skin
3	Fluorescent dye: Ethanol (1:1)	Paper/Laminate: H–VH (slightly < undiluted)	Paper/Laminate: H	Overall: M; brighter than heavier dilutions (1:2–1:4)
4	Fluorescent dye: Ethanol (1:2)	Paper/Laminate: M	Paper/Laminate: M	Overall: L–M
5	Fluorescent dye: Ethanol (1:3)	Paper/Laminate: L–M	Paper/Laminate: L–M	Overall: L
6	Fluorescent dye: Ethanol (1:4)	Paper/Laminate: L (faint)	Paper/Laminate: VL–L	Overall: VL; barely detectable on light surfaces
